# Impact of COVID-19 Pandemic Declaration on New Oncology Trial Commencements: An Interrupted Time Series with Segmented Regression Analysis

**DOI:** 10.3390/healthcare10030489

**Published:** 2022-03-07

**Authors:** Hyeon Uk Bin, Sohyun Jeong, Heeyoung Lee

**Affiliations:** 1 Department of Clinical Medicinal Sciences, Konyang University, Nonsan 32992, Korea; nabin1996@naver.com; 2 Marcus Institute for Aging Research, Hebrew SeniorLife, Harvard Medical School, Boston, MA 02131, USA; sohyunjeong@hsl.harvard.edu; 3 Department of Medicine, Beth Israel Deaconess Medical Center and Harvard Medical School, Boston, MA 02131, USA

**Keywords:** COVID-19, pandemic, oncology, clinicaltrial.gov

## Abstract

This study aimed to assess the trend in oncology trial commencements registered on ClinicalTrials.gov and to evaluate the contributing factors by comparing the trends in the pre- and post-COVID-19 pandemic era. The ClinicalTrials.gov database was searched to identify oncology study trials starting from 1 January 2018 to 28 February 2021. Data on the variables of start/complete date, phase, status, funding source, center, country and study type were extracted. According to the time point of the COVID-19 pandemic declaration by the World Health Organization (WHO), March 2020, we analyzed the extracted data, including interrupted time series (ITS) analysis and multivariable regression analysis. We identified 18,561 new oncology trials during the study period. A total of 5678 oncology trials in the prepandemic period and 6134 in the postpandemic period were included in the comparative analysis. The year 2020 had the most newly launched trials (32.3%), and the majority of trials were planned to be conducted for longer than two years (70.3%). The results of ITS show the trend in the commencement of oncology trials was significantly increased after the pandemic declaration (coefficient = 27.99; 95% CI = 19.27 to 36.71). Drug intervention trials were the largest contributor to the increased trial number compared to different interventions, such as trials of devices or procedures (OR = 1.14; 95% CI = 1.03 to 1.26, OR = 1.09; 95% CI = 0.91 to 1.29, and OR = 1.12; 95% CI = 0.96 to 1.31, respectively), whereas the United Kingdom was the highest contributor to the number of decreased trials (OR = 0.67; 95% CI = 0.51 to 0.89 *p* = 0.01) in the postpandemic era. The interruption in oncology trial initiation was diminished shortly after the COVID-19 pandemic declaration, which was influenced by several factors, such as interventions or national responses. Based on the current outcomes, appropriate strategies for developing oncology trials can be planned to mitigate the impact of future crises on oncology trials.

## 1. Introduction

The current outbreak of coronavirus disease 2019 (COVID-19) has become a major threat to public health [1]. Considering its severity and rapid global transmission in a short period, the World Health Organization (WHO) declared the COVID-19 pandemic on 12 March 2020 [2]. Within a year, the virus spread to many countries. Over 422 million cases have been confirmed, and around 5.8 million infected people have died as of 20 February 2022 [3]. The pandemic affected the lives of many people worldwide and exerted pressure on the global economy and health care system, including clinical research [4].

Clinical research requires high-quality evidence to guide clinical practice and advance public health [5]. For certain subgroups of patients with critical illnesses, such as cancer, finding optimal preventive and therapeutic alternatives over the course of treatment through clinical trials is crucial [6]. In particular, the impact of COVID-19 seems to be more significant for cancer patients [7,8]. Undertreatment caused by delay in the development of new cancer diagnoses or therapies could be one of the most critical issues for patients with cancer who are susceptible to infection with immunosuppression and/or coinciding side effects with COVID-19 symptoms [8]. However, while therapeutics and vaccines for COVID-19 were being actively developed, a decrease in translational research on “non-urgent” illnesses, including cancer, was observed at the beginning of the pandemic [9]. Compared with the prepandemic period, the COVID-19 pandemic was associated with a decrease in the number of new oncology clinical trials for drugs and biologic therapies by 60% on a global commercial clinical trial platform [10,11]. The decrease in oncology trials consequently caused delays in cancer and affected the COVID-19-related survival rate in cancer patients [12]. However, the trends in oncology trials are recovering in some areas, depending on the impact of COVID-19 on healthcare access and infrastructure [9,13,14]. Chen et al. reported a strong correlation between initial outbreak responses, such as the spread of infectious diseases, and public health capacity variations among countries [15], and regulatory restrictions and variations in implementing trials across countries have enabled oncology trials to return to prepandemic levels [12]. With such discrepancies, many existing studies have strived to investigate trends and factors influencing oncology trials in the initial era of the pandemic to plan future directions. Previously, Lamont et al. and Unger et al. also reported on new oncology trial commencement after the COVID-19 pandemic, but analyzing different data over a short period of time with discrepant outcomes might require more supportive evidence showing the impact of COVID-19 on the commencement of oncology trials [11,16]. As one of the first studies to investigate the trends and factors associated with the number of new oncology trials influenced by the COVID-19 outbreak followed by comparing the pre- and postpandemic era, the current study provides more comprehensive outcomes with trial information recorded during longer periods of time and affected factors to promote oncology trials during this medical crisis.

## 2. Materials and Methods

### 2.1. Data Source and Eligible Study

ClinicalTrials.gov website was searched using the word “Cancer” in the study results field and concurrently searched with synonyms, “neoplasm”, “tumor”, “malignancy”, “oncology”, “neoplasia”, “neoplastic syndrome”, and “neoplastic disease”, which were downloaded with all matching records as excel and XML files, and all relevant data are publicly available at www.clinicaltrials.gov (accessed on 18 October 2020). As one of primary registries of the WHO trial platform, providing more than 120,000 clinical research studies conducted in more than 175 countries, clinical trial information provided by ClinicalTrials.gov was downloaded [17]. To obtain standardized or consistent information on clinical trials, data from ClinicalTrials.gov were analyzed for the comprehensive understanding of oncology trials [18].

These records were searched on October 2020 when the number of COVID-19 confirmed patients was highest globally after pandemic declaration, and expectation of vaccine use was also significantly increased with first provision of guideline for vaccine use [19]. Eligible trials for inclusion of had start dates between 1 January 2018 and 28 February 2021. In addition, both observational and interventional studies were included regardless of the study status, such as active, recruiting, withdrawn, or complete. If trial information on study location, status, condition, intervention, funding source, clinical sites, and start date could not be found, these trials were excluded. In addition, clinical studies examining vaccines or treatment for COVID-19 were excluded to prevent cross-referencing. Additional information related to COVID-19, such as date of lockdown, nonessential retail reopening, and vaccination supply in each country, was extracted from open website source [20]. The number of clinical trials started before (referred to as the prepandemic era) and after the COVID-19 pandemic declaration (referred to as the postpandemic era) was compared. The date of March 2020 was used to define the pre- and postpandemic period based on the WHO declaration of the COVID-19 pandemic [21]: prepandemic period (from 1 March 2019 to 29 February 2020) and the postpandemic period (from 1 March 2020 to 28 February 2021). Each period comprised 1 year for even comparisons.

### 2.2. Study Variables

We included the following information from the ClinicalTrials.gov database as study variables: (1) trial phase, i.e., phase 1, phase 2, phase 3, or others (including early phase 1, phase 1/2, phase 2/3, phase 4, and not applicable phase); (2) intervention type, including drugs, procedures, devices, or others (including radiation, diagnostic tests, behavioral, genetic, dietary supplement, combination, or others, and sample size less than 5% of total trials in intervention category); (3) country of study, including the United States, Canada, France, Italy, the United Kingdom, China, South Korea, or others (including countries other than the seven countries selected based on the list of highly ranked countries conducting clinical trials as per Clinicaltrials.gov); (4) type of center, including single center, multicenter, and centers not reported; (5) trial duration was categorized based on the period between the recorded start date and completion date, including <1 year, 1–2 years, 2–3 years, and >3 years; and (6) study type was classified as interventional or observational.

Trials classified as “others” were mostly initially categorized by ClinicalTrials.gov, and those with number of trials being less than 5 percent of total sample in each category were also grouped as “others” to ensure the focus was on the effects of the main variables. Regarding therapeutic use, oncology trials reported drugs and biological products that were categorized as drugs since, in Clinicaltrials.gov, trials with drugs as intervention also included biological products. Furthermore, procedures including surgery and medical devices were classified based on the dataset provided by ClinicalTrials.gov.

Funding sources were categorized as industry, government, academia, cofunded, or others, based on funder types, sponsors, or collaborators provided by ClinicalTrials.gov. Trials funded by the National Institutes of Health (NIH) or the US federal government were included and classified as governmental. Trials solely funded by universities or colleges were classified as academia. To categorize others as funding sources, trials funded by individuals or community-based organizations (referred as IC) were included. In addition, cofunded trials were classified as trials: (1) government, academia, and IC-funded, (2) government and IC-funded, and (3) industry and IC-funded.

### 2.3. Statistical Analysis

Categorical variables were described as frequencies and percentages. Interrupted time series (ITS) analysis was used to investigate trends in oncology trial initiation after the COVID-19 pandemic declaration (March 2020) with all datasets between 1 January 2018 and 28 February 2021. Our ITS models were used to explain and predict possible correlations between data points studied with segmented regression analysis. The analysis model was as follows:

Yt = β_0_ + β_1_Time_t_ + β_2_pandemic declaration + β_3_Time after pandemic + ε_t_

Y_t_ is the outcome variable, defined as the number of oncology trials conducted each month at time t from 1 January 2018. Time_t_ is a continuous variable indicating the time in months at time t from the start of the study period. Pandemic declaration is a dummy variable indicating whether the declaration has been implemented or not (1 means after declaration, 0 means before declaration). Time after pandemic declaration (March 2020) was a continuous variable indicating the time in months after COVID-19 pandemic declaration. In this model, β_0_ estimates the baseline level of the outcomes, numbers of oncology trials per month at time zero. β_1_ estimates the change in the number of trials that occurred per month before pandemic declaration; β_2_ estimates the level of changes immediately after pandemic declaration; β_3_ estimates the changes in the trend of trial numbers after pandemic declaration.

To avoid time lag bias and recognize the declaration from WHO in clinical trial sites, trial records of the first month and three months after pandemic declaration were excluded to evaluate the trends of number changes in oncology trials, which was divided by three lags based on time periods. Lag 1 included overall trial records initiated from 1 March 2020 to 28 February 2021, and lag 2 was defined as the time period between 1 April 2020 and 28 February 2021 to exclude one-month data of trial records. Lag 3 included trial records excluding three-month data, and finally, trial records were included from 1 June 2020 to 28 February 2021 regarding time lag.

To compare one-year impact of COVID-19 after pandemic declaration to prepandemic period, chi-square test was used to assess the statistical significance of categorical variables. The test was two-sided, and significance was set at *p*-value < 0.05.

We then used a multivariate logistic regression model to identify the factors independently associated with the commencement of oncology trials between the pre- (from 1 March 2019 to 29 February 2020) and postpandemic (from 1 March 2020 to 28 February 2021) eras to investigate the relationship between oncology trial initiation and the explanatory variables, which included country, type of intervention, funding source, type of center, and study type. For binary response Y and a vector of explanatory variables X, the multivariate logistic regression model is given by:

Y = β_0_ + β_1 × 1_ + β_2_X_2_+ β_3_X_3_+ β_4_X_4_ + β_5_X_5_

Y is the probability of oncology trial initiation, and X is as follows.

X_1_ = country, X_2_ = type of intervention, X_3_ = funding source, X_4_ = type of center, and X_5_ = study type; to ensure the focus was on the effects of the main variables, the reference group was determined.

In addition, sensitivity analysis was performed with only trials showing “active, not-recruiting” as their status, defined as ongoing trials in ClinicalTrials.gov, compared to trials with different statuses, such as completed, withdrawn, terminated, and suspended. To evaluate the correlation of ongoing trials with other studies with different statuses, Pearson correlation coefficient (R) was analyzed. Independent odds ratios (ORs) and 95% confidence intervals (CIs) were estimated in this study. IBM^®^ SPSS^®^ Statistics 27 was used for all the statistical analyses.

## 3. Results

### 3.1. Basic Characteristics of Included Trials

**Figure 1:**
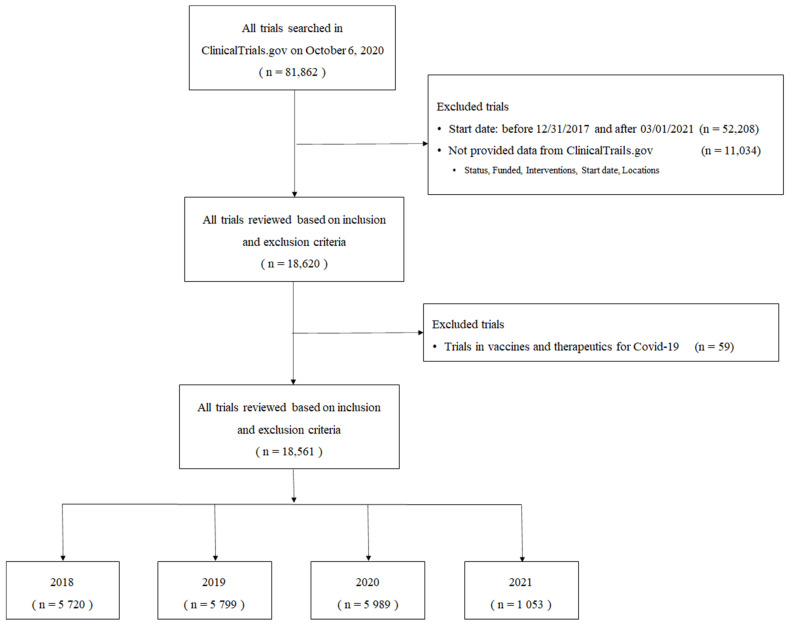
Flow chart of trial selecting oncology trials.

A total of 81,862 trials were identified without a time limit. A total of 29,654 trials were conducted between 1 January 2018 and 28 February 2021. After reviewing the information of the trials, such as the start and complete dates of studies, phase, country, status, location, study types, and intervention types collected with inclusion and exclusion criteria, 11,034 trials were excluded. Therefore, 18,561 trials were left for further analysis. For the 1-year comparison, trial records of 5678 oncology trials in the prepandemic period and 6134 in the postpandemic period were collected. The trials that started before 31 December 2017 and after 1 March 2021 were excluded (Figure 1). The characteristics of the included trials are presented in Table 1. The year 2020 had the most reported oncology trial commencements compared to the previous years (2018; 5720, 2019; 5799, and 2020; 5989).

Trial durations of >3 years were most often recorded (46.6%) compared to other study durations, such as <1 year (10.0%), 1–2 years (19.6%), and 2–3 years (23.7%). Interventional trials (80.3%) were more common than observational studies (19.7%). From 1 January 2018 to 28 February 2021, the percentage of phase 2 (23.4%) was high, while that of phase 3 (6.9%) studies was low. In addition, the status of studies was different, and the majority of oncology studies reported their study status as recruiting (59.1%). The countries with the highest number of trials were the US (32.1%), followed by China (17.0%), France (6.0%), Canada (2.5%), Italy (2.2%), the United Kingdom (2.2%), and South Korea (2.2%). The proportion of industry-funded trials was 19.7% of the total sample, followed by academia (18.8%), and government-funded trials (1.9%). Regarding the intervention types, drug trials (53.9%) were the most common, followed by procedures (7.5%) and devices (5.6%) (Table 1).

### 3.2. Overall Monthly Trends in the Launch of New Oncology Trials

**Figure 2:**
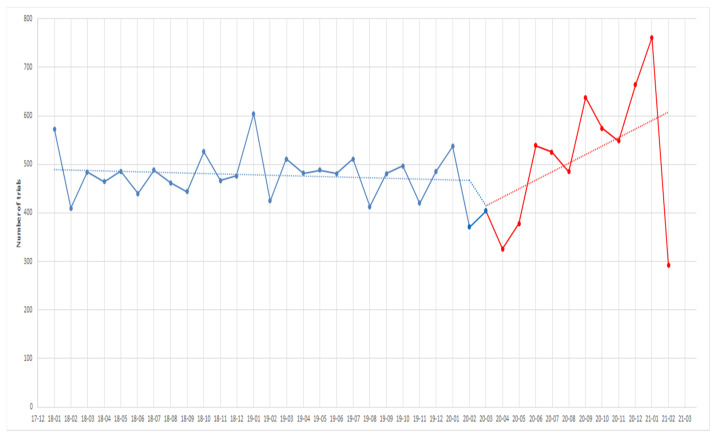
Monthly trends in the commencement of new oncology trials from 1 January 2018 to 28 February 2021.

The numbers and start dates of oncology trials conducted every month from 1 January 2018 to 28 February 2021 are shown in Figure 2. During the overall period, the results of the segmented regression analysis show that the trend of oncology trial initiation was negative before 1 March 2020 (coefficient = −1.25; 95% CI = −5.28 to 2.78), and the trend of oncology trial initiation was increased after the pandemic declaration (coefficient = 27.99, 95% CI = 19.27 to 36.71), which is consistently shown to increase in equal increments after March 2020 in Figure 2. Additional analysis to evaluate the time lag bias for accepting pandemic declaration in clinical sites presented an increased trend of oncology trial initiation according to the extraction of trial records during the COVID-19 pandemic period (Table 2).

**Figure 3:**
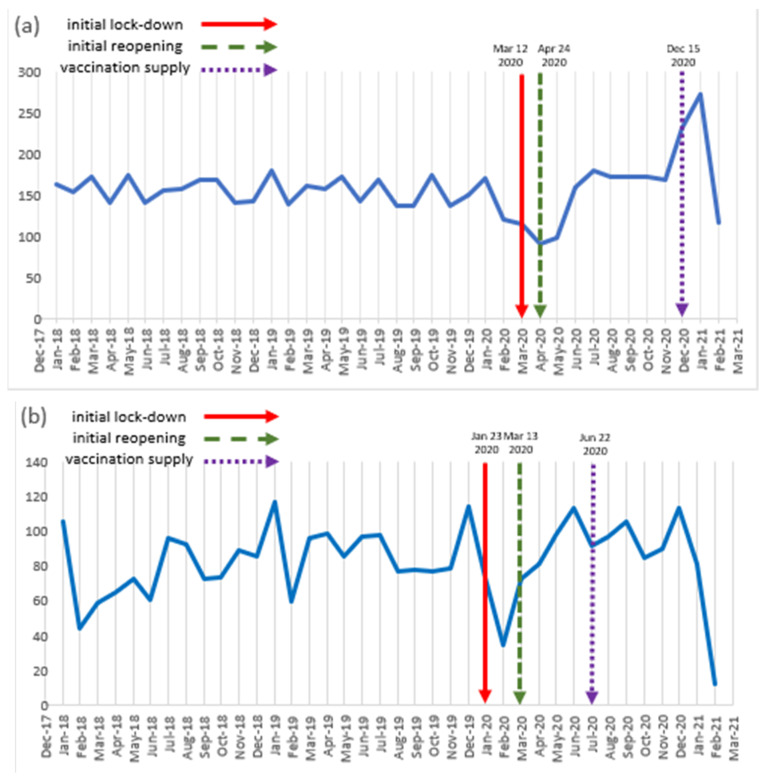

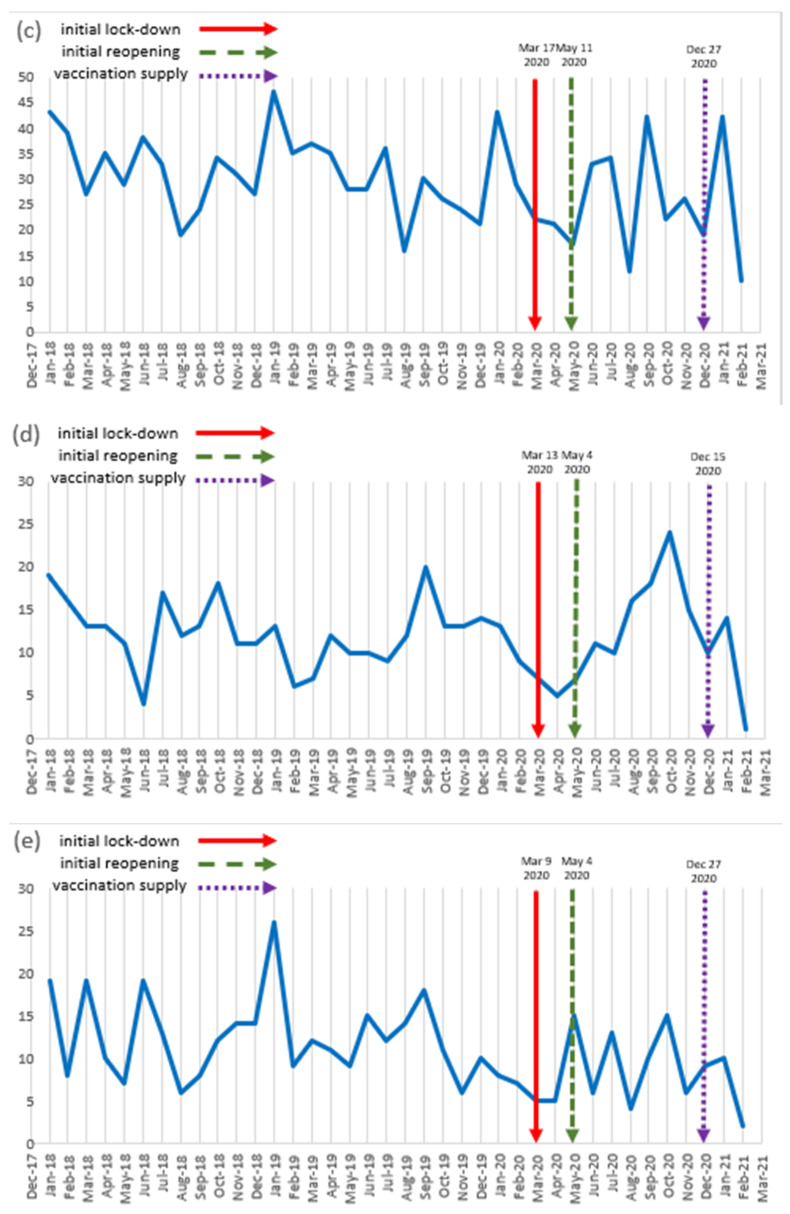

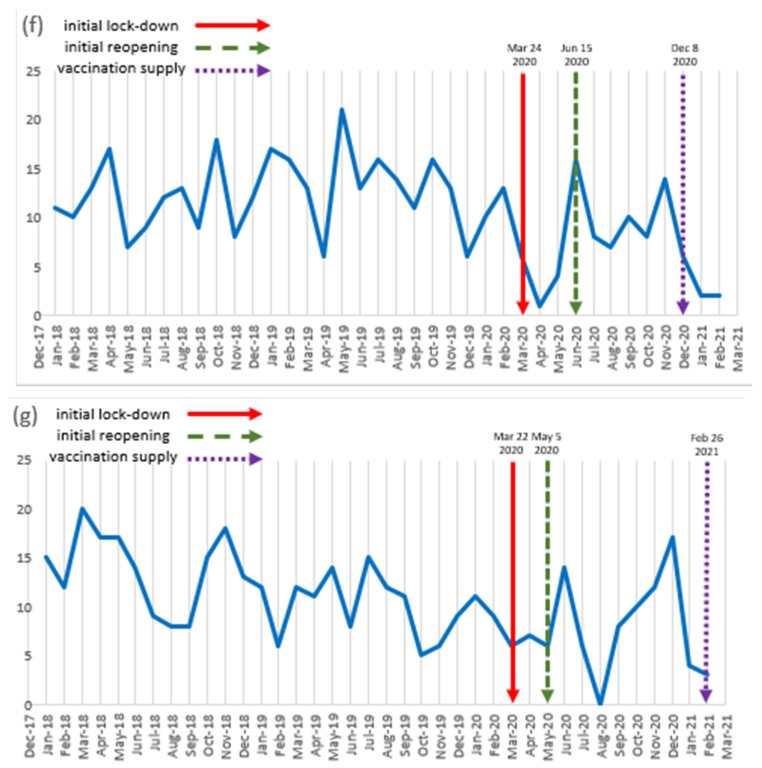
Monthly trends in the commencement of new oncology trials according to countries from 2018 to 2020. (**a**) United States; (**b**) China; (**c**) France; (**d**) Canada; (**e**) Italy; (**f**) United Kingdom; and (**g**) Korea.

Overall trends in the top seven countries are shown in Figure 3. Most countries showed a decline in the number of trials starting after the pandemic declaration on March 2020. Initial dates of lockdown and nonessential retail reopening in each country were different among countries, and there was a demonstration of a decrease in trial numbers except in Italy and South Korea.

**Figure 4:**
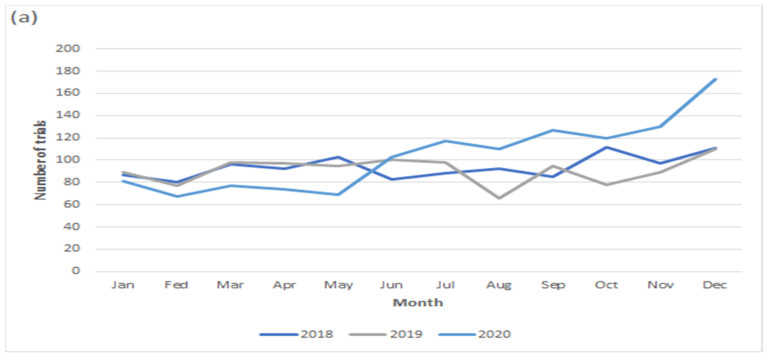

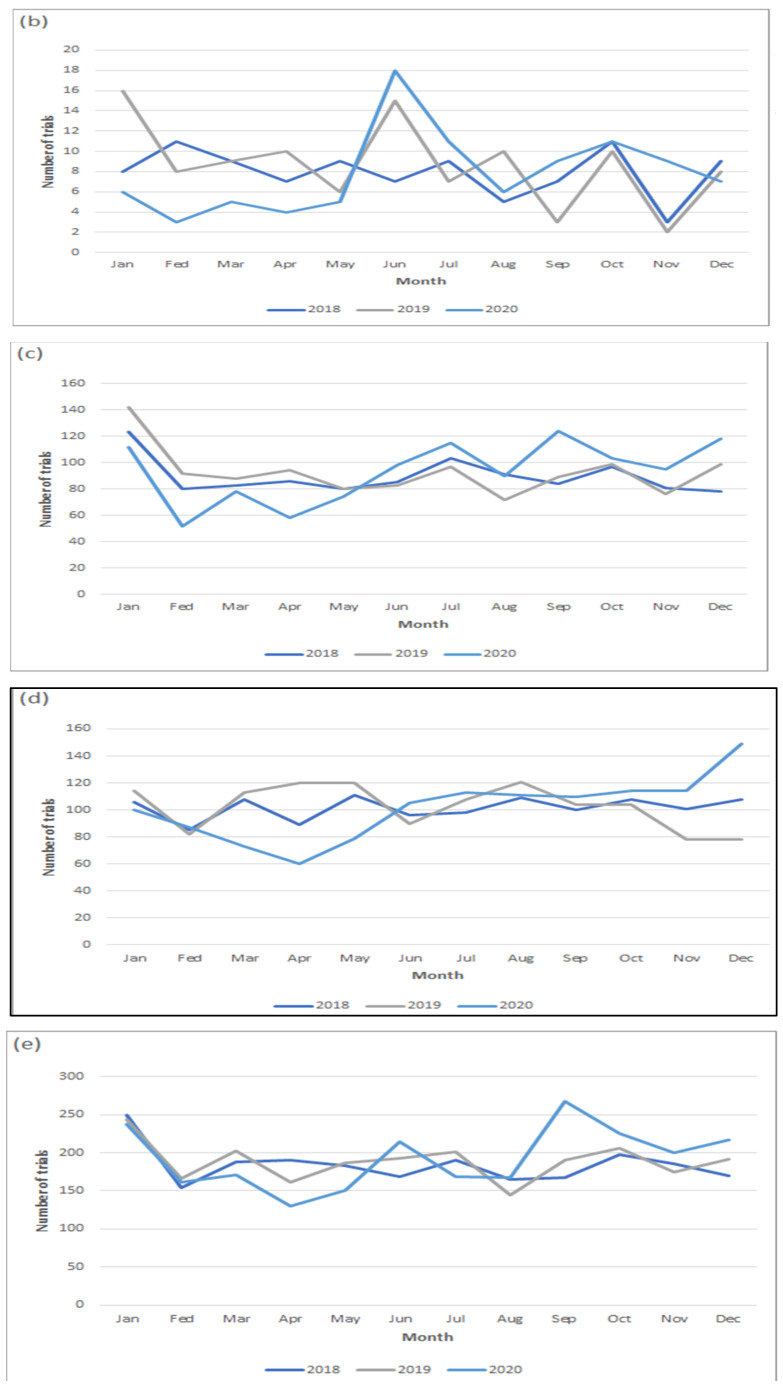
Monthly trends in the commencement of new oncology trials according to funding source from 2018 to 2020. (**a**) Industry; (**b**) government; (**c**) other; (**d**) academia; and (**e**) cofunded.

**Figure 5:**
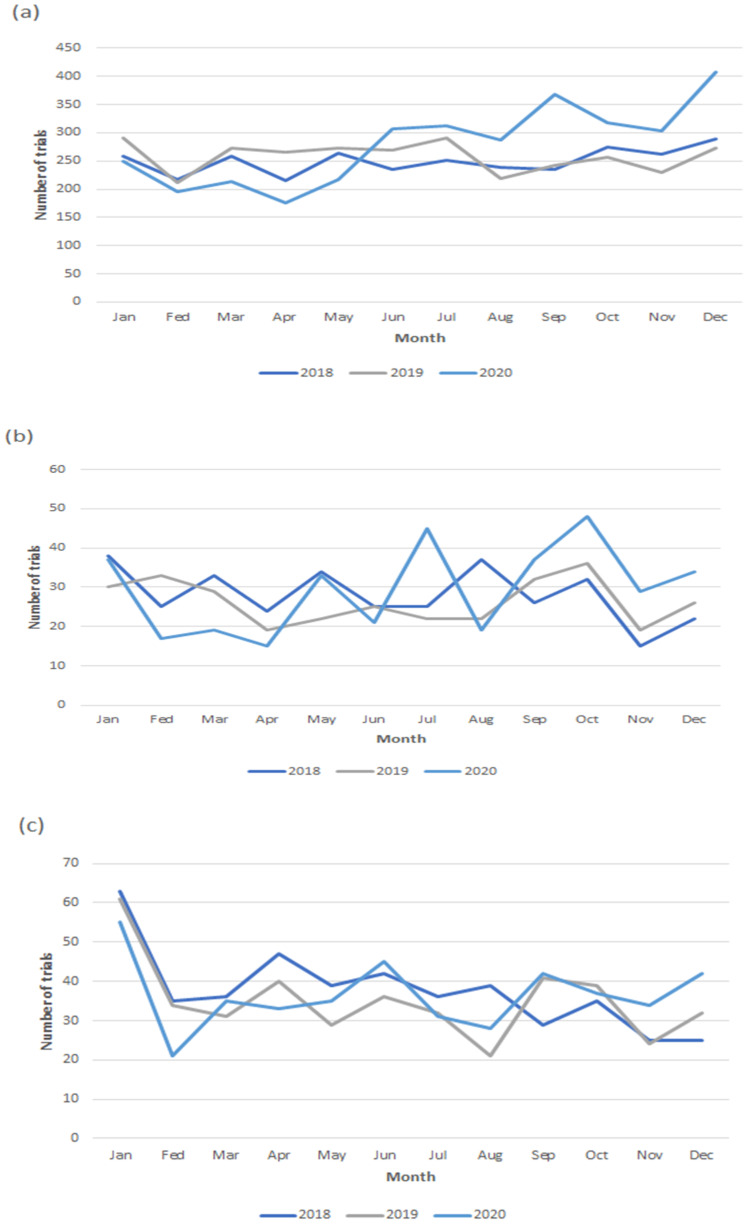
Monthly trends in the commencement of new oncology trials according to type of interventions from 2018 to 2020. (**a**) Drug; (**b**) device; and (**c**) procedure.

The number of trials was different based on funding sources, such as industry (19%), academia (18%), or government (1.2%) in March 2020, which showed an increased number of trials funded by the government (2%) and academia (1%) in June 2020, but other trial numbers were shown to be decreased in the same month, such as those funded by other organizations or those that were cofunded (Figure 4). The number of drug intervention trials showed similar trends to the previous two years (2018 and 2019) and presented an increased number of trials after the pandemic declaration compared to the previous years (Figure 5).

### 3.3. Comparisons of the Launch of New Oncology Trials in Pre- and Postpandemic Eras

We included 5678 oncology trials in the prepandemic period and 6134 in the postpandemic period for the 1-year pre- and postpandemic comparison analyses.

The overall trends in oncology trials conducted before and after the pandemic are shown in Table 3. Trial numbers based on phases for 1-year comparison between the pre- and postpandemic era were not significantly different (*p* > 0.05), which was consistently shown in trial numbers based on study type. However, trial numbers related to study status showed a discrepancy in the postpandemic era compared to the numbers in the prepandemic era, which showed the number of trials with all types of study statuses were decreased except for “not yet recruiting” (10.2% vs. 38.3%). Concerning funding sources, the proportion of trials funded by the industry (18.9% vs. 20.9%) and government (1.6% vs. 2.3%) increased in the postpandemic era. For type of centers, the proportion of trials conducted in multicenters (32.8% vs. 27.1%) and single centers (60.2% vs. 54.9%) was decreased in the postpandemic era.

In the country data, the US and China hosted the highest number of oncology trials in both periods; the US and China hosted 32.1% and 17.8% of the trials in the prepandemic period and 31.7% and 17.0% in the postpandemic period, respectively. In contrast, Italy (2.3% vs. 1.6%) and the UK (2.7% vs. 1.4%) showed a significant decline in the postpandemic period (*p* < 0.05).

### 3.4. Factors Affecting the Launch of New Oncology Trials in the Pre- and Postpandemic Eras

To analyze the factors affecting oncology trials, we performed a multivariate logistic regression analysis that included the study type, type of funding, type of center, country, and type of intervention (Table 4). Industry-funded and government-funded trials significantly contributed to the increase in oncology trials in the postpandemic era by 1.25 times (95% CI = 1.11 to 1.41; *p* = 0.00) and 1.44 times (95% CI = 1.08 to 1.91; *p* = 0.01), respectively, compared to “other” types of funding. Single-center trials showed a decreased number of new oncology trials (OR = 0.33; 95% CI = 0.28 to 0.38; *p* = 0.00).

The US and China were significant contributors to the number of increased trials at 1.21 (95% CI = 1.08 to 1.33; *p* = 0.01) and 1.14 (95% CI = 0.94 to 1.20; *p* = 0.04), whereas the UK contributed more to the decreased number of trials at 0.67 (95% CI = 0.51 to 0.89; *p* = 0.01) compared to other countries. Regarding the intervention type, drug trials (OR = 1.14; 95% CI = 1.03 to 1.26; *p* = 0.01) significantly contributed to the increase in oncology trials in the postpandemic era.

### 3.5. Sensitivity Analysis

The outcomes of the sensitivity analysis when comparing ongoing trials to other types of study statuses are shown in Table 5. The results show that the ongoing trial initiation number was positively correlated with several other types of study statuses, such as completed, suspended, terminated, withdrawn, and unknown statuses (*p* < 0.01). For the not yet recruiting status, the outcome was shown to be negatively correlated with ongoing trial initiation (R = −0.62; *p* < 0.01).

## 4. Discussion

The COVID-19 pandemic has created obstacles for the healthcare system and has considerably modified the care of cancer patients worldwide, affecting both patients and healthcare professionals [6,22]. Safety concerns regarding COVID-19 infections have hindered access to healthcare facilities. In particular, to minimize the need for additional laboratory examinations and follow-up visits, a suspension of clinical trials was frequently reported in the early COVID-19 era [9]. Although such situations are confirmed at some regional levels, other places have reported a minimal impact on clinical trials, despite the widespread use of COVID-19-related anxiety among investigators [9]. However, based on the current study, the trends in oncology trial numbers were observed after the pandemic declaration according to the outcomes of ITS analysis, which was consistently shown in other curves as well in the current analysis. In addition, an increase in the number of new oncology trials was also observed in the postpandemic era according to the 1-year comparison. Although a natural increase in the number of trials as the year went on could explain the number of new oncology trials during the postpandemic era, previous studies supported the finding that a natural increase in trial initiation did not occur [16,23]. Furthermore, in terms of the current outcomes, the number of new cancer trials varied among countries. Along with the national efforts against COVID-19, Upadhaya et al. [9] found that diverse situations related to oncology trials pushed the numbers back to the prepandemic level using investigator interviews in different countries or regions and that such diversity could influence the launch of oncology trials [2,9]. Considering that policy responses such as lockdown or social distancing contributed to reducing mortality and virus exposure, the policy response of each country to COVID-19 is one of the most important reasons for the use of healthcare systems and the sustained performance of clinical trials [15,24,25]. For starting oncology trials, accessibility to healthcare facilities or medical infrastructures to carry out processes, such as patient enrollment or monitoring, is important. Thus, national differences in policy responses to COVID-19 might have affected the number of trials being initiated [2,15,26]. Moreover, since differences in the implementation of new technologies or approaches to innovating clinical trial procedures, such as virtual trials or direct-to-patient delivery, have been implemented in some countries, this diversity could also be attributed to updating regulatory frameworks that positively affected the launch of new oncology trials [12].

In addition, after the relevant time period for accepting the pandemic declaration in clinical sites [27], the number of studies funded by both the industry and government increased in the postpandemic era compared to the prepandemic period. To sustain clinical trials, a stable funding source should be supported [28]; however, most resources have been directed to combat the COVID-19 crisis [29]. The current study findings indicate that funding for oncology trials by industries might be associated with the increased launch of new cancer trials during the pandemic [16], which explains their ongoing efforts to support and conduct oncology trials, as the industry hopes to proactively overcome the COVID lockdown situation [23]. Furthermore, although governments only included NIH or U.S. federal agencies, compared to the prepandemic era, in the postpandemic period, the number of cancer studies funded by the government increased.

As the intervention type, sample size, and completion period of clinical trials could vary according to the funding source and since the governments mostly fund early-phase of trials, such as phase 1 and/or phase 2 trials [30,31], the increase in phase 2 trials might be related to the increasing number of studies funded by the government in the postpandemic era. In addition, study types, such as interventional or observational studies, might be different depending on the funding source, and trend changes based on study types were not observed when comparing pre- and postpandemic periods in the current analysis. Although Gresham et al. reported a decline in NIH-funded trials from 2005 to 2015 [5], during the COVID-19 pandemic, government-funded medical research bodies in many countries were reported to maintain funding continuity for research studies [32]. Such an increase in the allocation of funds could influence the number of new oncology trials launched during the COVID-19 pandemic. While financial rescue cannot be the sole marker of sustained oncology research efforts [6], in the face of the COVID-19 crisis, sustained funding systems that provide emergency support and stable institutional standing to guarantee the continuity of new oncology trials are needed [5,23,29].

After the COVID-19 outbreak, cancer trials conducted with drug interventions continuously increased without significant fluctuations, as opposed to trials involving other types of interventions, such as procedures or devices. Oncology trials with drug interventions showed an increase from the pre- to postpandemic period. In the initial outbreak of COVID-19, although many cancer research studies were paused because the risk outweighed the benefits of starting trials, the need for safe and effective therapeutics for cancer patients remained high [33]. Regardless, cancer trials evaluating de novo compounds require large sample sizes and are costly, and these factors may have led to a high risk during the COVID-19 pandemic [5,6,33]. However, the hurdles that must be overcome to find safe and effective cancer therapeutics could be resolved by clinical trials repurposing existing therapies [34]. Furthermore, with a low rate of approval of new drugs, a rapid spike in interest in drug repositioning has been found in more than 1000 publications in 2020 [35]. The drug repositioning approach hastens the drug discovery procedure and has been the focus of researchers in a wide range of scientific areas [36], which might influence the development of new cancer trials on drugs, as shown in the current analysis. Along with shifting interests and the investment in COVID-19 therapeutics [16], the low overall developmental costs and low risk of drug repositioning [35] might contribute to the continuation of drug investigations in oncology trials during the pandemic era. Considering that drug repositioning trials are mostly funded by not-for-profit organizations or government agencies [33], the increased number of cancer trials funded by the government, as shown in the current analysis, might also reflect the increasing interest in oncology trials on drug repositioning in the postpandemic era.

The current study has several limitations. First, our study did not include all the studies that are currently being performed because investigators and sponsors may register their studies in other registries, which might not have been included in the database we checked [18]. Thus, future studies could examine universal and standardized data from all registries. Second, some oncology trials may not have been included in the analysis if essential keywords were not included in their registration. Misclassification may have also occurred. However, the data we used were screened by two independent investigators to minimize the risk of bias. Third, the current study did not update current records provided by ClinicalTrials.gov. However, the sponsor or principal investigator can update, correct, or delete information from the trial records of ClinicalTrials.gov at any time [18]. Without updated trial information, the current study still showed the trends of oncology trials at a time of medical crisis with the rapid spread of the virus and vaccination supply initiation. Fourth, the current study did not show the effect of industry funding sources on the increase in new oncology trials in the postpandemic era compared to the impact of nonindustry funding. In particular, obscure methodological approaches to defining the terms reported in Clinicaltrials.gov might cause diverse determinations related to the categories of variables in the analysis [37]. Thus, we expect that more future studies will be conducted with different datasets to compare the impact of industrial and nonindustrial funding sources. Fifth, the current study did not evaluate the effect of COVID-19 on the delay in trial commencement. Since there is no way to determine from ClinicalTrials.gov whether a trial commencement was delayed, we expect that more updated information related to the exact number of delayed trials will be provided and analyzed in the future. Sixth, the current study did not assess the COVID-19 outbreak severity based on country-level differences. Although the current study evaluated the impact of COVID-19 on oncology trial initiation, the relationship between the initial number of oncology trials and confirmed cases or deaths caused by COVID-19 in the context of outbreak severity was not assessed. However, this is beyond scope of the current study and may be evaluated in future studies. Seventh, the current study did not include information on funding sources supported by non-US governments. In ClinicalTrials.gov, however, the government category only included the NIH or US federal government. Since a surge in investments for medical research in China despite a decrease in U.S. federal funds has been reported [38], to fully understand how we can intervene in the future, the different impact of funding sources, especially those of the governments of countries over the pandemic, should be understood. Thus, in future studies, we expect more funding sources provided by non-US governments to be included in the analysis. Finally, the current study did not assess the long-term effects of the pandemic on the process of conducting oncology trials. Thus, we expect future studies to be conducted on the impact of COVID-19 on cancer trials and the factors influencing these trials long after the pandemic.

## 5. Conclusions

The current study demonstrated the increased trends of oncology trials after the COVID-19 pandemic, representing oncology trial recommencement. Although the decline in new cancer trials was observed at different degrees in different countries, various national responses to COVID-19 might have contributed to an increase in the number of new oncology trials. By evaluating the current trends and factors influencing the initiation of oncology trials, as shown in the current study, appropriate policy response, funding, and strategies for developing medicine can be planned to mitigate the impact of future crises on oncology trials.

## Figures and Tables

**Table 1:** Basic characteristics of included trials.

	Number	Percent
Period		
January 2018 to December 2018	5720	30.8
January 2019 to December 2019	5799	31.2
January 2020 to December 2020	5989	32.3
January 2021 to February 2021	1053	5.7
Trial duration		
<1 year	1857	10.0
1–2 year	3645	19.6
2–3 year	4406	23.6
>3 year	8653	46.6
Study type		
Interventional	14,913	80.3
Observational	3648	19.7
Trial phase		
Phase 1	2485	13.4
Phase 2	4336	23.4
Phase 3	1279	6.9
Others	10,461	56.4
Study status		
Not yet recruiting	3154	17.0
Recruiting	10,970	59.1
Enrolling by invitation	292	1.6
Active, not recruiting	1208	6.5
Completed	1316	7.1
Suspended	136	0.7
Terminated	266	1.4
Withdrawn	499	2.7
Unknown status	720	3.9
Funding source		
Industry	3651	19.7
Government	347	1.9
Academia	3485	18.8
Cofunded		
Government–Academia–IC	495	2.7
Government–IC	806	4.3
Industry–IC	2614	14.1
IC	7163	38.6
Centers		
Multicenter	5813	31.3
Single center	10,905	58.8
Not-reported	1843	9.9
Country		
United States	5962	32.1
China	3147	17.0
France	1114	6.0
Canada	457	2.5
Italy	417	2.2
United Kingdom	408	2.2
South Korea	400	2.2
Others	6656	35.9
Intervention		
Drug	10,006	53.9
Procedure	1387	7.5
Device	1043	5.6
Others ^a^	6125	33.0

IC, individual or community-based organization. ^a^ Diagnostic tests, radiation, behavioral, genetic, dietary supplement, combination, or others, sample size less than 5% of total trials in intervention category.

**Table 2:** Segmented regression analysis to assess time lag after pandemic declaration.

Parameters	Lag 1		Lag 2		Lag 3	
	Estimates (95% CI)	*p* Value	Estimates (95% CI)	*p* Value	Estimates (95% CI)	*p* Value
Intercept	490.61	**<0.00**	490.97	**<0.00**	485.53	**<0.00**
	(423.84–557.39)		(422.25–559.69)		(412.97–558.10)	
Slope before pandemic (β1)	−1.25	0.26	−1.17	0.29	−0.37	0.44
	(−5.28–2.78)		(−5.46–3.12)		(−5.69–4.95)	
Slope in pandemic declaration (β2)	15.35	0.05	14.83	0.06	9.01	0.11
	(−3.97–34.66)		(−4.75–34.42)		(−5.94–23.95)	
Slope in post pandemic (β3)	27.99	**<0.00**	27.01	**<0.00**	22.98	**<0.00**
	(19.27–36.71)		(17.88–36.12)		(9.10–36.86)	

Lag 1: 1 March 2020~28 February 2021; Lag 2: 1 April 2020~28 February 2021; Lag 3: 1 June 2020~28 February 2021; significant results are shown in bold type.

**Table 3:** Comparison of trends of launching new oncology trials in pre- and postpandemic eras *.

Categories	Number of Oncology Trials		*p* Value
	Prepandemic (*n* = 5678) (*n*,%)	Postpandemic (*n* = 6134) (*n*,%)	
Trial phase			
Phase 1	779 (13.7)	852 (13.9)	0.82
Phase 2	1309 (23.1)	1529 (24.9)	0.06
Phase 3	399 (7.0)	418 (6.8)	0.67
Others	3191 (56.2)	3335 (54.4)	0.28
Study type			
Interventional	4539 (79.9)	5005 (81.6)	0.48
Observational	1139 (29.1)	1129 (18.4)	0.60
Study status			
Not yet recruiting	578 (10.2)	2350 (38.3)	**<0.00**
Recruiting	4090 (72.0)	3411 (55.6)	**<0.00**
Enrolling by invitation	107 (1.9)	83 (1.4)	**0.02**
Active, not recruiting	300 (5.3)	94 (1.5)	**<0.00**
Completed	296 (5.2)	64 (1.0)	**<0.00**
Suspended	53 (0.9)	23 (0.4)	**0.00**
Terminated	68 (1.2)	4 (0.1)	**<0.00**
Withdrawn	154 (2.7)	105 (1.7)	**0.00**
Unknown status	32 (0.6)	0 (0.0)	-
Funding source			
Industry	1074 (18.9)	1285 (20.9)	**0.02**
Government	89 (1.6)	139 (2.3)	**0.01**
Academia	1041 (18.3)	1139 (18.6)	0.79
Cofunded Government–Academia–IC	155 (2.7)	173 (2.8)	0.77
Government–IC	266 (4.7)	278 (4.5)	0.71
Industry–IC	802 (14.1)	826 (13.5)	0.37
IC	2251 (39.6)	2294 (37.4)	0.09
Centers			
Multicenter	1863 (32.8)	1662 (27.1)	**<0.00**
Single center	3419 (60.2)	3366 (54.9)	**0.00**
Not reported	396 (7.0)	1106 (18.0)	**<0.00**
Country			
United States	1825 (32.1)	1944 (31.7)	0.71
China	1010 (17.8)	1041 (17.0)	0.33
France	353 (6.2)	300 (4.9)	**0.00**
Canada	142 (2.5)	138 (2.2)	0.38
Italy	133 (2.3)	100 (1.6)	**0.01**
United Kingdom	152 (2.7)	84 (1.4)	**<0.00**
Korea	123 (2.2)	93 (1.5)	**0.01**
Others	1940 (34.2)	2434 (39.7)	**0.00**
Intervention			
Drug	3036 (53.5)	3471 (56.6)	0.07
Procedure	306 (5.4)	338 (5.5)	0.78
Device	401 (7.1)	440 (7.2)	0.83
Others ^a^	1935 (34.1)	1885 (30.7)	**0.01**

* Pre and post were defined as of March 2020. IC, individual or community-based organization. ^a^ Diagnostic tests, radiation, behavioral, genetic, dietary supplement, combination, or others, sample size less than 5% of total trials in intervention category. Significant results are shown in bold type.

**Table 4:** Factors associated with the launch of oncology trials in postpandemic era as per the multivariable logistic regression model.

Category	Odds Ratio	95% CI		*p*-Value
		Lower Limit	Upper Limit	
Study type				
Interventional	1.01	0.91	1.12	0.87
Observational	1 ^a^			
Funding source				
Industry	1.25	1.11	1.41	**0.00**
Government	1.44	1.08	1.91	**0.01**
Academia	1.03	0.93	1.15	0.56
Cofunded				
Academia–Government–IC	1.10	0.87	1.40	0.42
Government–IC	1.02	0.85	1.24	0.81
Industry–IC	1.01	0.89	1.14	0.88
IC	1 ^a^			
Centers				
Mulitcenter	0.27	0.24	0.32	**0.00**
Single Center	0.33	0.28	0.38	**0.00**
Not reported	1 ^a^			
Country				
United States	1.21	1.09	1.34	**0.01**
China	1.14	1.01	1.28	**0.04**
France	1.07	0.90	1.27	0.47
Canada	1.16	0.91	1.49	0.23
Italy	0.93	0.71	1.23	0.62
United Kingdom	0.67	0.51	0.89	**0.01**
Korea	0.86	0.65	1.14	0.31
Others	1 ^a^			
Intervention				
Drug	1.14	1.03	1.26	**0.01**
Device	1.08	0.91	1.29	0.38
Procedure	1.12	0.96	1.31	0.14
Others ^b^	1 ^a^			

IC, individual or community-based organization. ^a^ Reference value. ^b^ Diagnostic tests, radiation, behavioral, genetic, dietary supplement, combination, or others, sample size less than 5% of total trials in intervention category. Significant results are shown in bold type.

**Table 5:** Sensitivity analysis comparing ongoing trials to other types of statuses.

Study Status	R ^a^	*p* Value
Not yet recruiting	−0.62	**<0.01**
Recruiting	−0.24	0.16
Enrolling by invitation	0.01	0.96
Completed	0.96	**<0.01**
Suspended	0.46	**<0.01**
Terminated	0.81	**<0.01**
Withdrawn	0.59	**<0.01**
Unknown	0.91	**<0.01**

^a^ Pearson correlation coefficient. Significant results shown are in bold type.

## Data Availability

All data are available on request to the corresponding author.
